# The relationship between achievement motivation and college students’ general self-efficacy: A moderated mediation model

**DOI:** 10.3389/fpsyg.2022.1031912

**Published:** 2023-01-04

**Authors:** Na Li, Ying Yang, Xiang Zhao, Yue Li

**Affiliations:** ^1^School of Physical Education, Shandong University of Science and Technology, Qingdao, China; ^2^School of Physical Education, Huaibei Normal University, Huaibei, China

**Keywords:** achievement motivation, general self-efficacy, perceived social support, sports participation, college students

## Abstract

**Objective:**

This study focused on the relationship between achievement motivation and college students’ general self-efficacy, and aimed to explore the mechanism of action between achievement motivation and general self-efficacy.

**Methods:**

Through convenience sampling, 1,076 college students were investigated from Anhui Province in China. Achievement motivation, general self-efficacy, perceived social support, and sports participation were evaluated using standard scales. For data analysis, Pearson’s correlation analysis, structural equation model test, and bias-corrected percentile Bootstrap method were carried out.

**Results:**

Common method biases can be accepted in this study. (1) Achievement motivation can directly affect general self-efficacy and make a positive prediction; (2) Perceived social support plays a mediating role between achievement motivation and general self-efficacy, that is, achievement motivation can indirectly affect general self-efficacy through perceived social support; (3) Sports participation plays a moderating role in the first half of the mediating path of “achievement motivation → perceived social support → general self-efficacy.” The interaction between achievement motivation and sports participation affects perceived social support, and then indirectly affects general self-efficacy. In this moderated mediation model, The predictive effect of achievement motivation on perceived social support is significantly different among individuals with different levels of sports participation.

**Conclusion:**

Perceived social support plays a part of mediating role between achievement motivation and college students’ general self-efficacy, which is moderated by sports participation.

## Introduction

The opinions on Further Strengthening and improving college students’ Mental Health Education issued by the Ministry of Education and others proposed that we should vigorously carry out college students’ mental health education, promote the healthy growth of college students, and cultivate high-quality and qualified talents (The Ministry of Education, the Ministry of Health, and the Central Committee of the Communist Youth League of the People’s Republic of China, 2018). College students are a special social group that has not yet graduated from college and entered the society. As a frontier group of new technologies and new ideas in society, and senior professionals trained by the state, the physical and mental health of college students has been the focus of attention from all walks of life. However, in the post epidemic period, college students are facing various pressures such as academics and employment, which lead to certain problems in their mental health and generally low self-efficacy.

Self-efficacy refers to people’s confidence in whether they can use their own skills to complete a certain work behavior (Bandura, 1978). It is a very important psychological variable and has an important impact on emotional intelligence (Feng et al., 2022), anxiety (Qingsong, 2022), depression (Zhiguang et al., 2021), and so on. The immune system function and mental health index of people with strong self-efficacy are also at good levels (Bandura, 1989a,b). General self-efficacy is a self-efficacy that does not depend on the domain (Sari and Bayazit, 2017). It reflects the belief and self-confidence level of an individual to take appropriate actions to deal with environmental challenges, and has a certain impact on the degree of effort in activities and the endurance and perseverance in the face of difficulties, setbacks and failures (Schunk and Pajares, 2009). People with high general self-efficacy believe that they are more capable of doing things well. Achievement motivation is an important subject of research in the field of psychology in recent years. Motivation is the dynamic system that affects students’ learning. The achievement motivation of college students is the main motivation that affects their learning. The formation of achievement motivation plays an important role in their own development. Achievement motivation is an internal driving force for individuals to pursue excellence and success. The higher the achievement motivation of college students, the greater the achievement in their career (Xia and Lirong, 2017).

In the context of health for all, the value of sport and the benefits it brings are valued. Students gain satisfaction and perceive a lot of social support when playing sports. The self-efficacy of adolescents to engage in physical activity is influenced by several factors, and social support is one of the most important ones (Feltz and Magyar, 2006). There is a strong and stable association between physical activity and self-efficacy (Higgins et al., 2014). By combing through a large amount of literature, physical exercise plays its functions of strengthening the body and regulating bad emotions, and the completion of challenging sports during exercise enhances individual’s perceived social support (Beets et al., 2010). Another study shows that social support enhances general self-efficacy (Shangjian, 2016). It is presumable that perceived social support and sports participation may have an influence effect on the relational pathway of achievement motivation and general self-efficacy to be further verified.

Some scholars have explored the close relationship between achievement motivation and general self-efficacy. Students’ achievement motivation and self-efficacy are in a cyclical and reciprocal relationship (Yan, 2022). Self-efficacy is an important cognitive resource that is positively and positively facilitated by an individual’s achievements (Majer, 2009). College students’ need for achievement can enhance self-efficacy (Yanru et al., 2022). Stress can increase self-efficacy by stimulating achievement motivation and decrease self-efficacy by inhibiting achievement motivation (Hao and Lichao, 2021). Dan et al. (2022) stated that the higher the achievement motivation, the more likely they were to choose challenging tasks, and their general self-efficacy was stronger. However, these studies have not deeply dissected the mechanism of the interaction between the two. Research on the relationship between achievement motivation and general self-efficacy can provide evidence-based support for the importance of improving college students’ general self-efficacy from a psychological perspective. Therefore, this study aims to deeply explore the relationship and mechanism between achievement motivation and college students’ general self-efficacy, in order to provide theoretical basis and practical guidance for enhancing college students’ general self-efficacy.

## Theoretical basis and hypothesis

### The relationship between achievement and general self-efficacy

Bandura, an American psychologist, first proposed the concept of “self-efficacy” in 1977. When an individual believes that he has the ability to complete a certain activity, he will have a high degree of “self-efficacy” and strive to complete this activity. Self-efficacy is usually regarded as a concrete expression of an individual’s self-confidence in a certain activity. It is a concept in a specific domain, that is, a person has a high degree of self-confidence in one aspect, but not necessarily in other aspects. However, German clinical and health psychologist Schwarzer and others believe that there is a general sense of self-efficacy, which is the overall self-confidence that individuals have when facing various new things and challenges in different environments (Schwarzer et al., 1997). General self-efficacy affects or determines a person’s behavioral choice, as well as his persistence and effort to the behavior. A person who believes that he can handle all kinds of things well will be more active and proactive in life. The higher the general self-efficacy level of college students, the higher the motivation to learn, and the more able they are to devote themselves to and focus on their academic (Kriegbaum et al., 2015).

The achievement motivation theory proposed by McClelland believes that achievement motivation refers to the self-driving force formed by outstanding individuals in the competition of survival of the fittest. It is the motivation for individuals to hope to engage in activities that are important and challenging to them, to achieve excellent performance and good results in the activities, and to catch up with and surpass others (McClelland, 1961). McClelland believes that those with high achievement motivation are more eager to succeed and more likely to succeed than those with low achievement motivation. Bandura’s self-efficacy theory of achievement motivation points out that the perception of competence (self-efficacy) of an individual determines the strength of his or her behavioral motivation in the achievement situation (Bandura, 1997). People with strong self-efficacy are more motivated to solve problems in adversity. Overcoming difficulties also reflects that they have high efficacy, thus maintaining a high level of achievement motivation. Students with this motivation factor can work hard in a difficult environment, overcome all kinds of difficulties and obstacles in learning, and achieve excellent results.

The study found that there was a significant positive correlation between general self-efficacy and achievement motivation. The stronger the general self-efficacy, the higher the achievement motivation. On the contrary, the higher the achievement motivation, the stronger the general self-efficacy (Yun et al., 2019). Although there is a certain correlation between achievement motivation and general self-efficacy, the process of how achievement motivation affects general self-efficacy is still unclear. Therefore, this study further explores the mediating and moderating mechanisms in the process of influence. Firstly, hypothesis H1 is proposed: achievement motivation directly affects and positively predicts general self-efficacy.

### The mediating role of perceived social support

Social support is an individual’s subjective perception and understanding of external support (Sarason et al., 1991). It is the spiritual and material help and support given by all aspects of society, including family, relatives, friends, colleagues, etc. Social support reflects the degree of closeness of the individual’s ties to society. Social support is roughly divided into two types: one is objective, actual or tangible support, including material direct help, and the other is subjectively experienced support, that is, perceived social support. Cullen believes that perceived social support refers to the emotional experience or satisfaction that an individual feels respected, supported and understood in society, which is closely related to the individual’s subjective feelings (Cullen, 1994).

Judging from the existing research results, there is a close relationship between perceived social support, achievement motivation and general self-efficacy. The social support college of students has a significant role in promoting achievement motivation. The higher the degree of social support, the stronger the achievement motivation, and social support can directly affect achievement motivation (Liru et al., 2018). There is a significant positive relationship between perceived social support and self-efficacy. Students with a high level of perceived social support have higher general self-efficacy; Similarly, the stronger the general self-efficacy of an individual, the deeper the social support he or she perceives (Yuqin, 2019). The improvement of perceived social support can correspondingly increase self-efficacy. Therefore, this study introduced perceived social support as a mediating variable and verified whether it played a partial mediating role in the path of achievement motivation affecting general self-efficacy, and proposed hypothesis H2: perceived social support plays a mediating role in the path of achievement motivation affecting general self-efficacy.

### The moderating role of sports participation

Sports and physical health are inextricably linked. Exercise can accelerate the metabolism of the body, promote the secretion of sebaceous glands, and sweat glands of the skin, and promote the discharge of wastes from the skin, so as to achieve the effect of improving skin quality. Endorphin produced during exercise is a substance with strong sedative effect, which can produce hypnotic effect, thereby reducing insomnia symptoms and having calming effect. Moreover, exercise is closely related to body temperature change, especially after aerobic exercise, the minimum body temperature can be lower than before. The lower the body temperature, the more sleepy the person is. Therefore, it is easier to fall asleep after exercise and sleep quality is better. Psychologists believe that physical exercise with appropriate load can promote the release of endorphins, allowing people to obtain a pleasant and exciting emotional experience, so as to relieve bad emotions and reduce stress. Therefore, participating in physical exercise, especially the sports that they love and are good at, can enable people to enjoy fun, boost spirits, reduce stress and maintain a good emotional state while strengthening their physical health (Chao and Guoli, 2020).

Under the background of the construction of “Healthy China,” the physical health of college students has been attached importance to. Exercise can regulate the mental state, make the exercisers happy physically and mentally, and reduce anxiety. Psychologist Bandura put forward the ternary interactive determinism for “whether behavior is determined by internal or external factors” (Bandura, 1989b), that is, individual, environment and behavior interact with each other. This study speculates that general self-efficacy, an individual cognitive factor, may interact with daily physical activity behavior. In addition, study has proved that self-efficacy is closely related to physical exercise (Kehong et al., 2021). Based on this background, hypothesis H3 is put forward: sports participation plays a moderating role in the effect of achievement motivation on general self-efficacy.

### The mediating effect of perceived social support and moderating sports participation

Achievement motivation, general self-efficacy, perceived social support and sports participation are closely related. Social support has a significant impact on college students’ physical exercise behavior and general self-efficacy. Social support and self-efficacy have a positive impact on college students’ physical exercise behavior. Self-efficacy has a positive effect on college students’ physical exercise behavior. The enhancement of self-efficacy can improve the persistence and persistence of college students’ physical exercise behavior and improve their physical quality and motor skills (Yakun et al., 2020). The higher the scores of social support and self-efficacy, the more able to promote the physical exercise behavior of adolescents (Kangfeng, 2021).

There is a significant positive correlation between the amount of physical exercise and the general self-efficacy of college students. With the increase of the amount of physical exercise, the self-efficacy is enhanced (Jianguo et al., 2016). Moreover, physical exercise is significantly related to self-efficacy. College students who regularly participate in sports activities have higher self-cognitive evaluations, have more confidence in handling and solving problems, and generally have stronger self-efficacy (Qin et al., 2017). While those with high self-efficacy are more willing to participate in and adhere to physical exercise (Ling et al., 2013). Improving self-efficacy can effectively improve the pleasure level of individuals when participating in sports, overcome the frustration in the process of sports participation, and then obtain better emotional regulation ability (Jianjun et al., 2016), and young people with positive attitude and high self-attitude are more likely to form the intention of participating in physical exercise (Hagger et al., 2001). In the context of physical education learning, goal orientation has a significant positive predictive effect on self-efficacy. By setting clear sports goals for students, it can enhance students’ pursuit of success motivation and self-confidence, thereby improving their self-efficacy.

**FIGURE 1:**
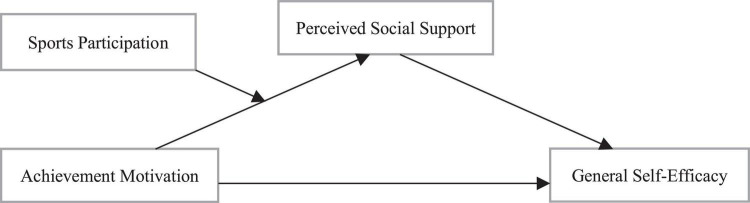
Schematic diagram of hypothetical moderated mediation model.

To sum up, this study intends to construct a moderated mediation model to investigate the mediating (perceived social support) and moderating (sports participation) mechanisms of achievement motivation in predicting the general self-efficacy of college students, so as to clarify the relationship between achievement motivation and college students’ general self-efficacy (Figure 1). It provides theoretical guidance for guiding college students to improve their achievement motivation and general self-efficacy.

## Materials and methods

### Participants and procedure

By using Achievement Motivation Scale (AMS), General Self-efficacy Scale (GSES), Perceived Social Support Scale (PSSS) and Sports Participation Questionnaire, a total of 1,076 freshmen and sophomores of public sports in four colleges and universities in Anhui, Zhejiang and Hebei were surveyed. College students were tested to explore the mediating and moderating effects between achievement motivation and college students’ general self-efficacy. Because junior and senior students have stronger motivation for employment or postgraduate entrance examination, it is not suitable for overall status assessment. Therefore, only freshmen and sophomores are selected for investigation, which is more conducive to accurately explore the relationship between achievement motivation and general self-efficacy. A total of 990 valid questionnaires were recovered, with a recovery rate of 92%, including 443 boys, 547 girls, 511 freshmen, and 479 sophomores.

### Materials

#### Achievement Motivation Scale

Norwegian psychologists T. Gjesme and R. Nygard compiled the AMS in 1970. In 1988, Chinese scholar Renmin Ye and Norwegian scholar K.A. Hagtvet jointly translated the Chinese version and revised it in 1992 (Renmin and Hagtvet, 1992). The scale is divided into two dimensions, pursuit of success and avoidance of failure. There are 30 questions in total. Its internal consistency coefficient is 0.83 (motivation to pursue success) and 0.84 (motivation to avoid failure). In this study, the whole scale’s Cronbach’s α and validity are 0.95 and 0.96, respectively, with good reliability and validity. The Cronbach’s α of pursuit of success and avoidance of failure are 0.92 and 0.90, respectively, and the validity are 0.94 and 0.92, respectively. According to the Likert grade 4 scoring method, 1 point is given for complete conformity and 4 points are given for complete non-conformity. The total score of items 1–15 is Ms (motivation to pursue success), and the total score of items 16–30 is Maf (motivation to avoid failure). The total score of the whole scale is Ma = Ms-Maf. The higher the score, the stronger the achievement motivation displayed.

#### General Self-Efficacy Scale

The GSES was compiled by Schwarzer et al. (1995). The Chinese version was revised by Caikang et al. (2001) and its good reliability and validity were verified. The Cronbach’s α of the scale is 0.94, and the validity is 0.93 in this study. The evaluation results have high reliability and validity. GSES is a one-dimensional scale with 10 items. With Likert’s grade 4 scoring method as the scoring standard, 1–4, respectively, indicate completely incorrect, slightly correct, mostly correct and completely correct. The sum of the scores the 10 items divided by 10 is the total scale score. The higher the score, the higher the general self-efficacy.

#### Perceived Social Support Scale

The PSSS was compiled by Zimet et al. (1991) and Qianjin (2001) translated and revised the Chinese version. The scale is a social support scale that emphasizes individual self-understanding and self-feeling. It consists of 12 items, including three dimensions of family support, friend support and other support. It measures various social support perceived by individuals. The level of social support perceived by individuals is mainly reflected in their emotional experience and satisfaction with respect, support and understanding. In this study, the Cronbach’s α of family support, friend support, other support and the whole scale are 0.90, 0.92, 0.87, and 0.95, respectively, and the validity are 0.83, 0.81, 0.82, and 0.94, respectively, showing good reliability and validity. The scale adopts the scoring method of Likert grade 7 evaluation, with 1 point for extremely disagree and 7 points for extremely agree. The total score is accumulated by the scores of all items, and it reflects the total degree of social support felt by the individual. The total score between 12 and 36 is a low support state; The total score between 37 and 60 is an intermediate support state; The total score is between 61 and 84 is a high support state. The higher the total score, the higher the degree of social support perceived by the individual.

#### Physical Activity Rating Scale-3

The Physical Activity Rating Scale-3 (PARS-3) was used. PARS-3 was compiled by Hashimoto Gongxiong and revised by Deqing (1994). Sports participation is evaluated from three aspects: exercise intensity, exercise time and exercise frequency. In this study, the Cronbach’s α of exercise intensity, exercise time, exercise frequency and the whole scale are 0.82, 0.91, 0.89, and 0.90, respectively, and the validity are 0.85, 0.88, 0.90, and 0.88. The scale adopts the scoring method of Likert grade 5. The intensity and frequency from 1 to 5 are scored 1–5 points, respectively, and the time from 1 to 5 is scored 0–4 points, respectively. The score calculation formula is exercise volume score = intensity score × time score × frequency score. The highest score is 100 and the lowest score is 0. Evaluation criteria of exercise volume: below 19 points are fluctuation, 20–42 points are moderate exercise, and 43 points and above are high exercise.

### Data analysis

Designed and distributed questionnaires, collected valid questionnaires, exported and sorted out data, and used SPSS26.0 and AMOS24.0 statistical analysis software to analyze and process the data. The subjects signed an informed consent form before administering the test, and the main subjects received professional training and were all graduate students in psychology. The administration process took about 15 min and all questionnaires were collected on the spot. The recovered data were screened to remove invalid and missing data and entered into the data. The data were statistically analyzed using SPSS for common method bias test, descriptive statistical analysis and correlation analysis. Amos was used to perform validation factor numerator for the matured scales and the degree of fit of the collected data. The mediating effect of perceived social support between achievement motivation and general self-efficacy of college students (Model 4) and the moderating effect of sports participation in it (Model 7) were analyzed using the Process plug-in prepared by Hayes (2015), and finally the conclusions of the study were obtained.

## Results

### The present situation of college students’ participation in sports activities

According to the data of PARS-3, the sports events that college students participate in show diversified characteristics. However, some activities are concentrated and are the choices of most college students, such as walking, running and ball games. The physical exercise of the surveyed subjects is mainly low-medium exercise volume. Different from the high requirements for physical fitness and competition needs of the students majoring in physical education, the students majoring in public physical education in physical exercise with less intensity.

In order to understand the current situation of college students’ sports participation, an independent sample *t*-test was conducted (the test value of exercise time was 2, and the test value of intensity and frequency was 3). It can be observed from Table 1 that the exercise intensity, exercise time and exercise frequency were all significantly different from the test values (*P* < 0.01), that is, there was statistical significance in college students’ sports participation. Comparing the average values of the three dimensions of exercise intensity, exercise time and exercise frequency with the test values, it was found that the current situation of college students’ sports participation is at a medium level, in which the levels of exercise time and exercise frequency were high, while the level of exercise intensity was low. Moreover, the questionnaire data showed that most college students mainly focus on sports exercise activities of medium and small intensity.

**TABLE 1:** Single sample statistics and test.

	*t*	*M*	*SD*	Boot CI (95% confidence interval)	
				Lower limit	Upper limit
Exercise intensity	11.727**	2.56	1.168	–0.51	–0.36
Exercise time	8.755**	3.34	1.223	0.26	0.42
Exercise frequency	17.692**	3.75	1.331	0.67	0.83

*N* = 990.

**p < 0.01.

### Common method bias control and inspection

The data collection method of this study was to issue questionnaires, which were filled out by students according to their own actual conditions. Therefore, common method bias test is required (Hao and Lirong, 2004). Harman’s single factor method was used for calculation and test. The test result showed that the maximum factor variance interpretation rate is 23.6%, which did not reach the critical standard of 40%, which indicated that there was no serious common method bias in this study.

### Analysis of variance of the influence of demographic characteristics on each variable and its different dimensions

Because the AMS total scale score is obtained by subtracting the scores obtained from the two subscales, and the PARS-3 scale score is obtained by multiplying the scores obtained by the three subscales, the two scales’ scoring method is special and different from the common method of calculating the mean value. Therefore, only the sub-dimension comparison of these two scales was carried out when calculating the demographic characteristics. The results showed that demographic variables have an impact on college students’ general self-efficacy, achievement motivation, perceived social support and sports participation.

As shown in Table 2, there were significant differences in exercise intensity and exercise time between genders (*P* < 0.01), and the difference in exercise time between different grades was statistically significant (*P* < 0.05). The exercise time of sophomores was longer than that of freshmen; there was no obvious gender difference in the two dimensions of achievement motivation, but there was a very significant difference between different grades (*P* < 0.01). The sophomores scored higher than the freshmen, especially the motivation to avoid failure; there were significant differences in the score of friends support dimension in different genders (*P* < 0.05), while the perceived social support score was the sum of the scores of the three dimensions. Overall, there was no significant difference in gender and grade; there was a significant difference in general self-efficacy between genders (*P* < 0.01). The level of general self-efficacy of boys was significantly higher than that of girls.

**TABLE 2:** Demographic difference of each variable and its different dimensions.

	Male	Female	*t*	*p*	Freshman	Sophomore	*t*	*p*
Exercise intensity	3.00 ± 1.15	2.22 ± 1.06	10.976	0.000**	2.54 ± 1.15	2.59 ± 1.19	–0.628	0.53
Exercise time	2.67 ± 1.23	2.07 ± 1.16	7.763	0.000**	2.26 ± 1.24	2.43 ± 1.21	–2.185	0.029*
Exercise frequency	3.71 ± 1.34	3.78 ± 1.32	–0.748	0.455	3.69 ± 1.34	3.81 ± 1.33	–1.457	0.145
Pursuit of success	2.47 ± 0.70	2.44 ± 0.56	0.688	0.491	2.27 ± 0.46	2.64 ± 0.72	–9.499	0.000**
Avoidance of failure	2.53 ± 0.61	2.52 ± 0.46	0.06	0.952	2.42 ± 0.44	2.64 ± 0.59	–6.767	0.000**
Family support	5.07 ± 1.19	5.12 ± 1.10	–0.693	0.489	5.07 ± 1.14	5.12 ± 1.14	–0.704	0.481
Friends support	5.07 ± 1.12	5.22 ± 1.02	–2.208	0.027*	5.15 ± 1.11	5.16 ± 1.04	–0.196	0.844
Other support	4.84 ± 1.13	4.89 ± 1.04	–0.65	0.516	4.86 ± 1.10	4.87 ± 1.06	–0.124	0.902
Perceived social support	4.99 ± 1.06	5.07 ± 0.95	–1.269	0.205	5.03 ± 1.02	5.05 ± 0.99	–0.381	0.703
General self-efficacy	2.82 ± 0.65	2.45 ± 0.52	9.702	0.000**	2.63 ± 0.64	2.59 ± 0.58	1.185	0.236

*N* = 990.

*p < 0.05; **p < 0.01.

### Descriptive statistics and correlation coefficient

The correlation coefficient matrix showed (Table 3) that perceived social support, sports participation and achievement motivation are significantly positively correlated with general self-efficacy, with correlation coefficients of 0.265, 0.294, and 0.074, respectively. When the correlation coefficient is calculated, the closeness of the relationship cannot be judged simply by the magnitude of the correlation coefficient (Huping, 2015). The correlation coefficient between achievement motivation and general self-efficacy is small and weak, but there is still a correlation (*p* < 0.05), which means that the correlation is significant and statistically significant. As the sample size increases, the correlation coefficient also changes. So, it is not significant for us to consider the correlation coefficient alone, and it is more meaningful to consider the significance. This indicated that the higher the achievement motivation, the higher the general self-efficacy level, and hypothesis H1 was verified. There was a significant positive correlation between sports participation and perceived social support (*P* < 0.05), and the correlation coefficient was 0.110; There was no significant correlation between achievement motivation and perceived social support, and between achievement motivation and sports participation. The independent variable and the moderating variable were relatively independent, which is suitable for testing moderating effect.

**TABLE 3:** Descriptive statistics and correlation coefficient of each variable.

	*M*	*SD*	1	2	3	4
1. General self-efficacy	2.611	6.098	1			
2. Perceived social support	60.45	12.035	0.265**	1		
3. Sports participation	26.27	24.366	0.294**	0.110**	1	
4. Achievement motivation	–1.09	5.388	0.074*	–0.046	0.002	1

*N* = 990.

*p < 0.05; **p < 0.01.

The mean value of each variable was compared with the median value of the scale score. The minimum value of general self-efficacy was 1.0, the maximum value was 4.0, and the mean value was 2.611 > 2; The minimum value of perceived social support was 12, the maximum value was 84, and the mean value was 60.45 > 36; The minimum value of sports participation was 0, the maximum value was 100, and the mean value was 26.27 < 50; The minimum value of achievement motivation was –21, the maximum value is 24, and the average value was –1.09 < 0. To sum up, the overall general self-efficacy of the survey respondents was strong, and the level of perceived social support was generally high, but the level of sports participation was low, and the achievement motivation was also weak.

### Construction and test of the moderated mediation model

To investigate the relationship between several variables, a moderated mediation model was constructed, and the effects were tested. SPSS was used to standardize the scores of each variable, and the process plug-in was used to test the model effect. The specific operation method: firstly, build the mediation model and test its fitness and mediation effect. If there is mediation effect, then introduce a moderating variable, and use Model 7 to test the moderated mediation mode.

Amos was used to construct a structural equation model with perceived social support as the mediating variable, achievement motivation as the predictor variable, and general self-efficacy as the outcome variable to test whether perceived social function can play a mediating role in the relationship between the two. Based on Process model4 developed by Hayes, the hypothetical mediation model was tested.

From the fitting index of the model (Table 4), it can be seen that χ^2^/df = 1.810 < 3, RMSEA = 0.029 < 0.05, and GFI, AGFI, CFI were all greater than 0.9, all adaptation indicators were within the specified range. Therefore, it can be considered that the constructed mediation model has a good fit.

**TABLE 4:** Fitness index of structural equation model of achievement motivation.

χ^2^/df	RMSEA	GFI	AGFI	CFI	IFI	NFI
1.810	0.029	0.996	0.987	0.998	0.998	0.996

After the Bootstrap test (Table 5), the direct effect and the mediation total effect of the model were all significant (*P* < 0.01) and the confidence interval were within 95%. Achievement motivation can directly affect general self-efficacy, and it can also indirectly affect general self-efficacy through perceived social support. That is, the mediation model to be constructed was established, and perceived social support played a part of mediating role between achievement motivation and general self-efficacy. Assuming that H1 and H2 were verified, the mediating effect path is: achievement motivation—perceived social support—general self-efficacy.

**TABLE 5:** Mediation model effect.

	β	*t*	*p*	Boot CI (95% confidence interval)	
				Lower limit	Upper limit
	1.7982	18.8357	0.0000**	1.6108	1.9855
Achievement motivation	0.0098	2.8322	0.0047**	0.0030	0.0166
Perceived social support	0.0136	8.7828	0.0000**	0.0106	0.0167

*N* = 990.

**p < 0.01.

The interference factors that may affect the explanatory variables were controlled, and the regression analysis was carried out with gender and grade as the control variable, and the moderated mediation model was tested. To avoid the influence of the grade factor on the accuracy of the results, a separate regression analysis was conducted using the grade of the survey respondents as a control variable. It was found that the product of the interaction terms did not significantly predict the mediating variable, there was no moderating effect on this path, and the moderating effect of the mediating model was not significant. The regression analysis with gender as the control variable found significant effects for each path, and this moderated mediation model holds. Thus, sports participation had a significant moderating effect among college students of different genders, but it did not have a moderating effect among college students of different grades. The results of hierarchical regression analysis were shown in Table 6. Achievement motivation can directly affect and significantly positively predict general self-efficacy (β = 0.009, *P* < 0.01), perceived social support significantly positively predicted general self-efficacy (β = 0.014, *P* < 0.01), that is, there was a significant correlation and predictive relationship between the variables of the mediation model. Through the results of regression equation, it was found that the interaction of achievement motivation and sports participation had a significant effect on perceived social support, indicating that sports participation played a moderating role on the path of “achievement motivation—perceived social support.” R-Square of general self-efficacy in regression analysis was 0.173, the *F*-value was 68.925, and the R-square of perceived social support was 0.023, the *F*-value was 5.847.

**TABLE 6:** Results of hierarchical regression analysis.

	General self-efficacy				Perceived social support			
	β	*SE*	*t*	*p*	β	*SE*	*t*	*p*
	2.366	0.111	21.291	0.000**	54.859	1.671	32.834	0.000**
Achievement motivation	0.009	0.003	2.652	0.008**	–0.271	0.108	–2.500	0.013*
Sports participation					0.070	0.017	4.158	0.000**
Achievement motivation × sports participation					0.006	0.003	1.974	0.049*
Gender	–0.379	0.036	–10.665	0.000**	1.800	0.794	2.268	0.024*
Grade	–0.012	0.028	–0.424	0.672	0.518	0.615	0.842	0.400
Perceived social support	0.014	0.001	9.690	0.000**				
Sample size	990				990			
R-square	0.174				0.024			
Adjusted R-square	0.169				0.018			
F	51.695**				4.818*			

*N* = 990.

*p < 0.05; **p < 0.01. All variables in the model were standardized and then entered the regression equation.

**FIGURE 2:**
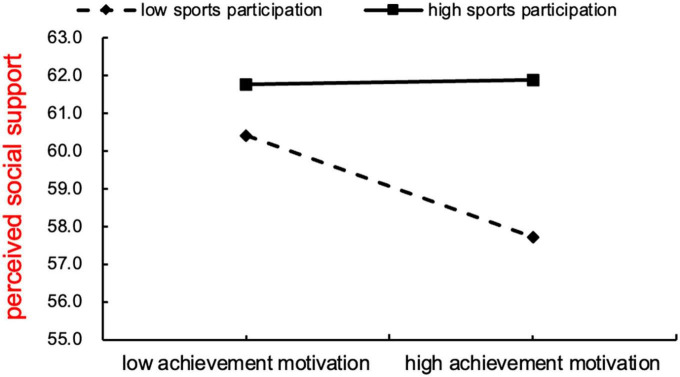
The moderating effect of sports participation on achievement motivation and perceived social participation.

After testing, sports participation had a moderating effect on the first half of the mediation model (Table 7), the simple slope was further used to analyze how sports participation moderated the path “achievement motivation—perceived social support.” As shown in Figure 2, the extended part of the two lines intersected, that is, there was a moderating effect, and the predictive effect of achievement motivation on perceived social support was significantly different among individuals with different levels of sports participation. When college students were at a low level of sports participation (M-1SD), achievement motivation had a strong negative impact on perceived social support (β = –0.252, *P* < 0.05), the perceived social support decreased with the improvement of achievement motivation; When college students were at a high level of sports participation (M + 1SD), achievement motivation had a positive effect on perceived social support, but it is not significant (β = 0.023), perceived social support increased with the improvement of achievement motivation. Therefore, sports participation can moderate the mediation model of “achievement motivation—perceived social support—general self-efficacy,” hypothesis H3 was verified.

**TABLE 7:** Moderating effect of different levels of sports participation.

Sports participation	β	Boot SE	*t*	*p*	Boot CI (95% confidence interval)	
					Lower limit	Upper limit
M-1SD	–0.252	0.104	–2.424	0.016*	–0.456	–0.048
M(24.366)	–0.114	0.071	–1.616	0.106	–0.254	0.025
M + 1SD	0.023	0.093	0.249	0.803	–0.159	0.205

*N* = 990.

*p < 0.05.

**FIGURE 3:**
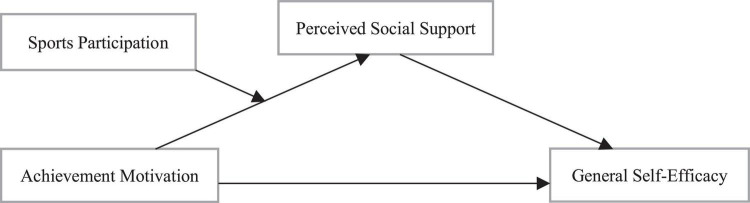
Schematic diagram of final model.

To sum up, the moderated mediation model constructed was established (Figure 3), and perceived social support played a partial mediating role between achievement motivation and college students’ general self-efficacy, and this mediating process was moderated by sports participation. In this moderated mediation model, the interaction of achievement motivation and sports participation significantly predicted perceived social support, and perceived social support significantly positively predicted general self-efficacy.

## Discussion

### The relationship between achievement and general self-efficacy

This study focuses on several psychological and behavioral factors of college students, investigates and explores the relationship between these factors, and concludes that there are mediating and moderating mechanisms in the process of achievement motivation affecting college students’ general self-efficacy.

There are significant differences in general self-efficacy between genders. Boys’ general self-efficacy is stronger, which may be related to factors such as gender roles, social expectations, social culture (Bingcheng, 2014). The gender characteristics and behavior patterns of men and women are different. It is generally believed that boys’ abilities are stronger than girls’ and they should bear more responsibilities than girls’ in society. This default expectation makes boys show stronger general self-efficacy. There is no significant difference in the general self-efficacy of the students of different grades, indicating that grades have little effect on general self-efficacy. There is no significant gender difference in achievement motivation, but there is a significant difference between different grades. Sophomores score higher than freshmen, especially the motivation to avoid failure. Under such motivation, tasks that are easy to complete and not challenging are often chosen. One study showed that achievement motivation levels were significantly higher in the upper grades than in the lower grades, and that achievement motivation increased with each grade level (Liangjie and Shuang, 2018). This is consistent with the findings of this paper, where sophomore students had higher achievement motivation scores than freshmen students. This may be due to the fact that freshmen students have not yet adapted to college life, while sophomores have adapted and gotten used to college campus life and have clearer plans for their future. Higher grade students are more internally motivated to learn, prompting them to exhibit stronger achievement motivation (Shuohao and Gaigai, 2019). Achievement motivation has a direct impact on college students’ general self-efficacy and can have a positive predictive effect on it. Whether it is the motivation to pursue success or the motivation to avoid failure, it is the starting point for individuals to successfully complete a certain task and achieve their own goals. The reason is that the achievement motivation is closely related to the individual’s self-confidence level (Chongzeng and Xiting, 2007), the need for achievement and the evaluation of self-cognition (McClelland, 1961). When an individual believes that he is capable of completing a certain activity or is competent for a job, he will have a high sense of “self-efficacy.” Only when he has a clear positioning of his own ability can he judge whether he is confident of successfully completing this task. Ability and self-confidence, as common elements of achievement motivation and general self-efficacy, to a certain extent support the existence of influence relationship between achievement motivation and general self-efficacy, which can be predicted.

Achievement motivation can affect college students’ general self-efficacy, which may be related to personal pursuit and learning status (Fang et al., 2013). College students are full of longing for the future. Whether it is employment or postgraduate entrance examination, students with high requirements or strong learning ability have strong self-confidence and high goals, and can be highly committed to personal goals and work hard for them. It may also be motivated by one’s own internal need for achievement, where efforts to reach achievement goals lead to improved judgments of one’s own abilities, thus enhancing general self-efficacy.

### The mediating role of perceived social support

The results show that there is significant gender difference in the score of friends support dimension, with girls scoring higher than that of boys. This may be related to the dependence of girls, who are more willing to talk to their friends and ask for help when encountering problems. Compared with boys, they prefer to be accompanied by others (Hua et al., 2018). The difference of perceived social support of grades is not significant, indicating that grades have little effect on college students’ perceived social support.

Through the research, it is found that achievement motivation can indirectly affect general self-efficacy through the mediating factor of perceived social support, and the fitting factors of the constructed “achievement motivation → perceived social support → general self-efficacy” mediation model all reach a good level, supporting the partial mediating effect of perceived social support between achievement motivation and general self-efficacy. The reason why social support plays a mediating role may be that achievement motivation reflects one’s own achievement needs and goal pursuit (Lan and Qin, 2022). Some studies have shown that perceived social support plays a partial mediating role in the path of self-efficacy affecting achievement motivation (Yaru et al., 2019), which again indirectly proves that mediating effect of perceived social support between self-efficacy and achievement motivation, and the two can have an indirect impact through the mediation of perceived social support. The mediating role of perceived social support between achievement motivation and general self-efficacy reveals that we can enhance general self-efficacy by enhancing perceived social support. Social support is the encouragement and approval of an individual’s abilities and behaviors from various aspects, and an individual’s sense of self-efficacy is enhanced when he or she is fully affirmed and motivated. When one’s achievement motivation is known by people around him, he will get support and encouragement from the surrounding, and the achievement motivation will be strengthened. The perceived social support reflects the total degree of social support that an individual receives subjectively. When an individual perceives that he or she is supported by different degrees from various aspects, self-confidence will be enhanced (Baodan et al., 2018), and the increase of self-confidence will lead to the improvement of general self-efficacy. Therefore, it is necessary to enhance the subject’s perceived support in social interactions, which in turn leads to the enhancement of general self-efficacy.

### The moderating role of sports participation

This paper investigates the current situation of sports participation of some college students. The health status is not high, and there are also insufficient sports and lack of physical exercise, especially for female students. And the enthusiasm of sports participation is low, the awareness of sports is weak, and the sense of identification with sports activities is low. There is no gender and grade difference in sports frequency, and the mean difference in sports time is small. Overall, there is little difference in sports participation between grades. There is a significant difference in the amount of physical activity between genders, and the amount of physical activity of boys is significantly greater than that of girls, which has also been verified in the research of Changjun et al. (2015). The test subjects of this study are mainly engaged in physical activities of medium and small intensity, with higher exercise time and frequency, and lower exercise intensity. The reason may be that the subjects of the survey are students of public sports. Their physical quality and athletic ability are lower than those of the students majoring in physical education, and the exercise load that the body can bear is also lower. The amount and intensity of load during exercise are controlled to prevent the body from being damaged. Compared with the students majoring in physical education, the students of public sports do not achieve the ideal level of sports through participating in sports activities, and do not gain a sense of physical and mental pleasure (Aifang, 2016). The research results show that the exercise intensity and time of male students are higher than that of female students. The reason is that the physical quality and athletic ability of male students are stronger than that of female students, and the sense of pleasure and achievement obtained through a large amount of exercise will also be higher than that of female students (Zhengge and Yuhui, 2022). However, many girls lack interest in sports and have low exercise intensity. They may be worried that high-intensity exercise will develop muscles and affect the beauty of body shape (Wen et al., 2021).

Pearson coefficient tested the correlation between achievement motivation and sports participation, and found that there was no significant correlation between them. Sports participation as a moderator modulates the first half of the pathway of achievement motivation on college students’ general self-efficacy, i.e., sports participation enhances the positive effect of achievement motivation on college students’ achievement motivation is stronger in individuals with high sports participation. The predictive effect of achievement motivation on college students’ general self-efficacy tended to increase significantly as the level of sports participation increased. The reason for the moderating effect of sports participation may be that, due to their high achievement motivation, the sense of achievement and pleasure obtained by college students participating in physical exercise can restrain their inner restlessness and anxiety (Chaohui, 2020), and enhance their subjective self-confidence through exercise. Perceived social support was significantly positively correlated with exercise time, exercise frequency and exercise intensity. The higher the level of sports participation, the higher the perceived social support (Zhihao et al., 2019). College students actively exert their potential in the process of exercise, get the encouragement and support from teachers and classmates, and increase the perceived social support, which can enhance their general self-efficacy. Improving the participation in daily physical exercise, overcoming difficulties and setbacks in sports can help improve exercise motivation (Wen and Beihe, 2020) and general self-efficacy (Jingtao et al., 2022). In sports, self-efficacy is mainly reflected in the belief and self-confidence to overcome a series of difficulties and participate in sports. Active and effective sports participation has a significant effect on improving the self-confidence level of college students (Chunmei et al., 2022). Sports participation as a form of social behavior affects the psychological state of the individual. As achievement motivation grows, students with high levels of sports participation have a faster growth in their level of appreciative social support and a stronger sense of general self-efficacy.

## Conclusion

(1) Achievement motivation is significantly positively correlated with general self-efficacy, which can be positively predicted.

(2) Perceived social support plays a partial mediating role between achievement motivation and general self-efficacy.

(3) Sports participation plays a moderating role in the relationship between achievement motivation, perceived social support and general self-efficacy.

The conclusions of this study reveal the influence mechanisms of achievement motivation on general self-efficacy. This model is constructed to hope that improve achieve motivation of college students can improve their general self-efficacy by actively participating in physical exercise and obtaining more social support. It is suggested that in order to enhance the general self-efficacy of college students, we need to pay attention not only to increase the level of achievement motivation but also to increase their level of sports participation. The enhancement of achievement motivation will have a positive impact on the general self-efficacy of individuals with high levels of sports participation compared to those with low levels of sports participation.

## Data availability statement

The original contributions presented in this study are included in this article/Supplementary material, further inquiries can be directed to the corresponding author/s.

## Ethics statement

The studies involving human participants were reviewed and approved by the Institutional Review Board of the School of Physical Education at Huaibei Normal University. The patients/participants provided their written informed consent to participate in this study.

## Author contributions

XZ and NL: conceptualization, investigation, writing–review and editing, project administration, and funding acquisition. NL, YL, and YY: methodology, formal analysis, and writing. NL, XZ, YY, and YL: writing–original draft preparation. All authors contributed to the article have read and agreed to the published version of the manuscript.
